# Evaluation of a real world intervention using professional football players to promote a healthy diet and physical activity in children and adolescents from a lower socio-economic background: a controlled pretest-posttest design

**DOI:** 10.1186/1471-2458-14-457

**Published:** 2014-05-16

**Authors:** Veerle Dubuy, Katrien De Cocker, Ilse De Bourdeaudhuij, Lea Maes, Jan Seghers, Johan Lefevre, Kristine De Martelaer, Hannah Brooke, Greet Cardon

**Affiliations:** 1 Department of Movement and Sport Sciences, Ghent University, Watersportlaan 2, B-9000 Ghent, Belgium; 2 Department of Public Health, Ghent University, De Pintelaan 185, B-9000 Ghent, Belgium; 3 Research Foundation Flanders, Egmontstraat 5, B-1000 Brussels, Belgium; 4 Department of Kinesiology, KU Leuven Tervuursevest 101, B-3001 Leuven, Belgium; 5 Department of Movement and Sports Training, VU Brussel, Pleinlaan 2, B-1050 Brussels, Belgium; 6 MRC Epidemiology Unit and UKCRC Centre for Diet and Activity Research (CEDAR), Institute of Metabolic Science, University of Cambridge School of Clinical Medicine, Cambridge Biomedical Campus, Box 285, Cambridge CB2 0QQ, UK

**Keywords:** Physical activity, Healthy diet, Football, School program, Health promotion, Disadvantaged children

## Abstract

**Background:**

The increasing rates of obesity among children and adolescents, especially in those from lower socio-economic backgrounds, emphasise the need for interventions promoting a healthy diet and physical activity. The present study aimed to examine the effectiveness of the ‘Health Scores!’ program, which combined professional football player role models with a school-based program to promote a healthy diet and physical activity to socially vulnerable children and adolescents.

**Methods:**

The intervention was implemented in two settings: professional football clubs and schools. Socially vulnerable children and adolescents (n = 165 intervention group, n = 440 control group, aged 10-14 year) provided self-reported data on dietary habits and physical activity before and after the four-month intervention. Intervention effects were evaluated using repeated measures analysis of variance. In addition, a process evaluation was conducted.

**Results:**

No intervention effects were found for several dietary behaviours, including consumption of breakfast, fruit, soft drinks or sweet and savoury snacks. Positive intervention effects were found for self-efficacy for having a daily breakfast (p < 0.01), positive attitude towards vegetables consumption (p < 0.01) and towards lower soft drink consumption (p < 0.001). A trend towards significance (p < 0.10) was found for self-efficacy for reaching the physical activity guidelines. For sports participation no significant intervention effect was found. In total, 92 pupils completed the process evaluation, the feedback was largely positive.

**Conclusions:**

The ‘Health Scores!’ intervention was successful in increasing psychosocial correlates of a healthy diet and PA. The use of professional football players as a credible source for health promotion was appealing to socially vulnerable children and adolescents.

## Background

Throughout the world the prevalence of overweight and obesity among children and adolescents has taken on epidemic proportions [1,2]. In general, higher rates of overweight and obesity are noted in European boys than in girls [3]. Furthermore, children and adolescents from low-income families are more likely to be obese than their counterparts from higher income backgrounds [4]. Both short term and long term consequences of childhood obesity have been established, including insulin resistance, sleep disorders, low self-esteem and an overall increased risk of adult morbidity and mortality [5].

Several complex and interacting processes are involved in the development of overweight and obesity [6,7]. Nevertheless, the main cause of obesity is a chronic energy imbalance, which occurs when energy intake exceeds energy expenditure [8,9]. Dietary intake and physical activity (PA) are important behaviours related to the energy balance [9,10] and are considered key elements in the prevention of overweight and obesity [11-13].

Concerns exist about the unhealthy dietary habits and low levels of PA in adolescents [14,15]. Gender differences in diet and PA can be observed: boys have higher soft drink consumption than girls, but a larger proportion of boys meet the PA guideline than girls [3]. Though, socio-economic disparities in these behaviours raise further concerns. Recent reviews have indicated that adolescents with lower socio-economic status (SES) perform less PA than those with higher SES [16,17]. With respect to nutrition, lower soft drink consumption and higher daily fruit intake has been shown in children and adolescents from higher socio-economic backgrounds [18]. Reducing these socio-economic inequalities in health behaviours is a major priority for public health policy in Europe [19].

Engaging the target population and successfully communicating the intervention message are essential elements for an effective intervention. The Elaboration Likelihood Model (ELM) is centred on persuasive communication [20] and the basic premise of the model is that there are two ways of processing information: a central and a peripheral route. In contrast to the central route, peripheral processing occurs when the motivation and ability to process a persuasive message is relatively low. Under these conditions, the acceptance of the information can be improved by providing information through peripheral cues. Peripheral cues can include the characteristics of the source of the information [21]. The use of role models, who appear successful and are perceived as credible by the target population, has been effective in prompting others to behave similarly [22]. Based on these findings, a local health promotion service developed an intervention using professional football players as a potentially credible source to promote a healthy diet and PA in socially disadvantaged children. Professional football players were chosen as in Flanders football is among the most popular sports in youth, especially in boys of lower SES [23]. About 40% of all boys between 10 to 18 years mention football as their favourite sport [24]. Also, similar projects abroad provide conservative evidence for the use of professional football players as a successful strategy to reach socially vulnerable groups [25,26]. Evidence of the positive effects of the use on celebrities or sports heroes to deliver persuasive messages largely exist in the field of marketing, this approach is less common for the field of health promotion [27,28]. Therefore, this real world intervention was used to explore the effectiveness of a school program combined with the use of professional football players as a credible source for promoting positive dietary habits and PA in socially disadvantaged children.

## Methods

### Intervention development and implementation

The ‘Health Scores!’ intervention was developed by a local-regional network for prevention in collaboration with the Football^+^ Foundation. The Football^+^ Foundation is an organisation that provides opportunities and financial support to Belgian professional football clubs to be involved in socially relevant projects. ‘Health Scores!’ was inspired by the Dutch intervention ‘Scoring with Health’ [25] and was based on the ELM [20]. The Dutch intervention promoted a healthy diet and PA in children from 9-12 years old, using professional football players as role models in combination with a school program. Similar to the Dutch intervention, the use of professional football players as a credible source for promoting health behaviours was the key intervention strategy and the program consisted of three components: (1) a start clinic, (2) a school program, (3) and an end clinic. The intervention had two main topics: (1) a healthy diet and (2) PA. From September 2011 to March 2012 local practitioners, including health workers and teachers, implemented the intervention in two settings: football clubs and schools.

The football clubs were responsible for organising the start and end clinics, which took place at the football club. During the clinics activities encouraging a healthy diet and PA took place (e.g. eating a healthy breakfast, a warm-up session with football players and signing a lifestyle contract handed out by a professional football player). At both clinics professional football players were involved in the activities and promoted these health behaviours.

Between the start and end clinics, a four-month school-based program took place. This comprised of school and class room activities connected to both intervention topics (e.g. providing free fruit to all pupils, a fruit and vegetable quiz, a lesson on the importance of drinking enough water, active playgrounds and activity breaks during lessons) To facilitate the implementation of the intervention, teachers received a tutorial consisting of a range of activities on a healthy diet and PA. As the use of professional football players was the key intervention strategy, two video messages (one on a healthy diet and one on PA) and two letters from the professional football players reminding the pupils of the importance of regular PA and a healthy diet were provided. These messages could be used as lesson introduction.

### Participants

The intervention was presented to each professional football club in Flanders, the Flemish speaking part of Belgium, (n = 8) by the Football^+^ Foundation. Seven football clubs were willing to participate. Together with the Centres for Student Counselling, each football club was responsible for the selection of the schools. Potential schools were ranked – based on official indicators [29] – according to the proportion of socially vulnerable pupils. The first schools on the list were contacted and invited to participate. Each football club could include as many classes of pupils as they wanted, with a minimum of 50 pupils. All pupils in the last two years of elementary school and the first two years of secondary school (10-14 year olds) were eligible. Depending on the number of eligible pupils per school, football clubs had to contact a number of schools.

A similar selection procedure was used by researchers to compile a control group, which received care as usual, including PE lessons as this is a mandatory component of the school curriculum. To optimize comparability with the intervention group, comparison schools were matched on region, school authority (catholic or community schools) and grade of the pupils (elementary of secondary school). A ratio of 1/3 control en 2/3 intervention pupils was aimed at.

### Instruments

The development of the questionnaire was based on two existing questionnaires [30,31]. Demographic information, including age, gender and employment status of both parents, was collected in the first part of the questionnaire. The second part assessed dietary habits and was based on the valid and reliable Food Frequency Questionnaire (FFQ) [30]. Consumption frequencies were assessed for fruits, vegetables, water, soft drinks and sweet and savoury snacks (response categories: ‘never’, ‘less than once a week’, ‘once a week’, ‘2-4 days/week’, ‘5-6 days/week’, ‘once a day, every day’, ‘every day, more than once’). Breakfast habits and psychosocial correlates of breakfast habits including attitude and self-efficacy were also assessed. Respondents were asked to report how many days in a week and a weekend they had breakfast. Attitude towards a daily breakfast was determined by asking the following question on a five point scale: ‘*Do you think you should have breakfast every day?’*. The question: *‘I find having breakfast every day….’* with the response categories ‘very difficult’, ‘difficult’, ‘simple’, ‘easy’ and ‘very easy’ was asked to determine self-efficacy towards a daily breakfast. The general attitude towards the above mentioned food items was also evaluated on a five point scale.

In the final part of the questionnaire, PA levels were determined using the Flemish Physical Activity Questionnaire (FPAQ). The FPAQ has an acceptable validity and a moderate to high reliability (>0.70) for the indexes used in the present study [31]. Activity habits were operationalized as 1) duration of active transport and 2) duration of sport participation. Active transport was assessed by the number of days per week respondents reported walking and cycling to and from school and the duration of each journey. To establish sports participation, the respondents reported their three main sports (total duration of participation). In addition, data on psychosocial correlates of PA were collected. Attitude towards PA was determined by asking the question: ‘*Do you like sports/PA?’*. The question: *‘I find one hour of PA a day…’* (response categories: ‘very difficult’, ‘difficult’, ‘simple’, ‘easy’ and ‘very easy’) was asked to determine self-efficacy towards PA.

At follow-up, pupils of the intervention group received additional questions to obtain process data. Pupils were asked to evaluate both clinics (activities during clinics and overall appreciation [1 (very dissatisfied) to 10 (very satisfied) scale]). They reported which topics had been discussed in class and whether they had seen the video messages and letters from the professional football players. The pupils’ appreciation of the messages and letters, as well as the program overall [1 (very dissatisfied) to 10 (very satisfied) scale], was also obtained.

### Procedure

Data were collected twice (baseline and follow-up) through self-administered questionnaires.

In both the intervention and control group a large proportion of the pupils did not speak Dutch in the home environment, so teachers and researchers clarified the meaning of questions where uncertainty arose. The most frequently reported countries of birth, besides Belgium, were Turkey, Morocco, Bulgaria, the Netherlands, Albania and the Czech Republic.

**Figure 1:**
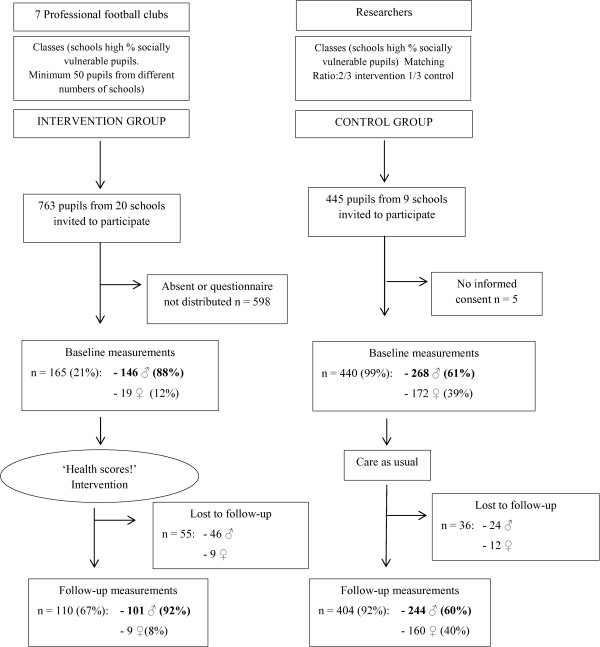
Flowchart of the study participants of the ‘Health Scores!’ intervention.

In the intervention group, measures were taken during the start clinic (September – November 2011) and four months later during the end clinic. In the control group, measurements took place in participating schools during the same time periods as the start and end clinics.In total, data from 165 children in the intervention group and 440 children in the control group were available. Initially 763 pupils in the intervention group were eligible for inclusion. However, at the start of the project there were no clear instructions for the distribution of the questionnaire among the intervention group. As a result several football clubs did not distribute the questionnaire among the pupils, which limited the data available. Data from 91 children were lost to follow-up due to absence at measurements, children changing schools or questionnaires filled out inaccurately (see Figure 1).

All parents of the pupils in the control group gave passive informed consent, which required parents to sign and return a form if they refuse to allow their child to participate. For pupils in the intervention group, passive informed consent was used for both the start and end clinic, the school program was incorporated into the regular curriculum. The study protocol was approved by the Ethics Committee of the Ghent University.

### Data analysis

All data were analysed using Statistical Package for the Social Sciences version 21.0 for Windows. Preliminary analyses consisted of descriptive statistics of sample characteristics. Drop-out analyses comparing baseline demographic and behavioural characteristics of pupils participating and not participating at follow-up were conducted. Demographic characteristics of intervention and control respondents were compared using independent-sample t-tests and chi-square tests for continuous and categorical variables respectively.

Repeated measures analyses of variance (MANOVA), with time (baseline/follow-up) as the within-subjects factor and condition (intervention/control) as the between-subjects factor, were used to evaluate the effects of the intervention on PA and dietary habits, and on related correlates. Descriptive statistics were used to summarise the process data. The level of statistical significance was set at p < 0.05, but p-values <0.10 were considered indicative of a trend towards significance.

## Results

### Sample characteristics

Preliminary analyses revealed a significantly higher proportion of girls in the control group (n = 172; 39%) than in the intervention group (n = 19; 12%) (X^2^ = 43.80 p < 0.001). Given the limited number of girls in the intervention group and because football is especially popular among boys, girls were excluded from all further analyses. This resulted in 146 and 268 pupils in the intervention and control group respectively.

Baseline demographic characteristics of the intervention group were compared with the control group (see Table 1). Pupils in the control group were somewhat younger than pupils in the intervention group. No significant difference in paternal employment status was found, but there was higher maternal employment in the control group than in the intervention group. Therefore, further analyses were controlled for age of the child and maternal employment status.

**Table 1:** Baseline demographic characteristics and baseline and follow-up values of dietary habits and PA for intervention and control groups

**Baseline comparability**					
	**Intervention group (n = 146)**	**Control group (n = 268)**	**X**^**2 **^**or t (p)**		
Age (years)	12.57 ± 1.02	12.08 ± 1.58	3.61 (***)		
Employment status father (% employed)	88%	84%	0.86 (ns)		
Employment status mother (% employed)	44%	62%	9.86 (**)		
**Intervention effects**					
	**Intervention group (n = 101)**		**Control group (n = 224)**		**Time by condition F (p)**
	**Baseline**	**Follow-up**	**Baseline**	**Follow-up**	
**Healthy diet**					
Attitude breakfast consumption (scale 1-5)	4.17 ± 0.98	4.11 ± 1.15	4.20 ± 0.87	4.37 ± 0.87	2.37 (ns)
Self-efficacy breakfast consumption (scale 1-5)	3.51 ± 1.01	4.17 ± 0.98	3.59 ± 1.09	3.61 ± 1.03	9.20 (**)
Breakfast consumption (days/week)	4.91 ± 2.30	4.96 ± 2.32	6.20 ± 1.67	6.23 ± 1.66	0.48 (ns)
Attitude vegetable consumption (scale 1-5)	3.60 ± 0.99	4.32 ± 0.85	3.49 ± 1.27	3.37 ± 1.37	8.84 (**)
Vegetable consumption (times/week)	3.32 ± 2.12	4.85 ± 2.26	4.06 ± 2.18	4.49 ± 2.08	**3.25 (#)**
Attitude fruit consumption (scale 1-5)	4.15 ± 1.06	4.28 ± 0.84	3.96 ± 0.92	4.17 ± 0.83	1.01 (ns)
Fruit consumption (times/week)	4.49 ± 2.46	4.28 ± 2.56	4.69 ± 2.44	4.21 ± 2.41	0.42 (ns)
Attitude soft drink consumption (scale 1-5)	2.17 ± 1.05	4.08 ± 1.03	2.49 ± 1.05	2.45 ± 1.28	32.61 (***)
Soft drink consumption (times/week)	4.42 ± 2.25	4.77 ± 2.54	3.22 ± 2.38	3.42 ± 2.46	1.55 (ns)
Attitude water consumption (scale 1-5)	2.66 ± 1.56	2.70 ± 1.58	2.93 ± 1.19	2.93 ± 1.37	0.26 (ns)
Water consumption (glasses/day)	2.47 ± 1.45	3.17 ± 1.34	2.85 ± 1.33	2.79 ± 1.30	**2.94 (#)**
Attitude sweet and savoury snack consumption (scale 1-5)	3.26 ± 1.33	3.58 ± 1.29	3.10 ± 1.11	2.85 ± 1.25	1.06 (ns)
Sweet and savoury snack consumption (mean pieces/week)	1.32 ± 0.91	1.25 ± 1.04	0.78 ± 0.89	0.76 ± 0.90	0.03 (ns)
**Physical activity**					
Attitude physical activity (scale 1-5)	4.48 ± 0.97	4.81 ± 0.39	4.96 ± 0.20	4.89 ± 0.36	0.84 (ns)
Self-efficacy physical activity (scale 1-5)	2.90 ± 1.34	4.15 ± 1.23	3.35 ± 1.33	3.30 ± 1.27	**2.86 (#)**
Sports participation (mean minutes/week)	158.02 ± 77.86	165.73 ± 72.95	167.82 ± 67.45	170.99 ± 67.90	1.36 (ns)
Active transport (minutes/week)	52.71 ± 66.61	66.08 ± 103.64	70.14 ± 76.63	80.76 ± 90.55	0.16 (ns)

Values are mean ± SD or %, ns = non-significant (p > 0.05). #trend towards significance; *0.01 < p ≤ 0.05; **0.001 < p ≤ 0.01; ***p ≤ 0.001.

Drop-out analyses showed few significant differences. However, pupils who participated at follow-up consumed significantly more fruit (p = 0.005) and had higher sports participation (p = 0.004) at baseline than pupils who did not participate at follow-up.

### Intervention effects

Data on dietary habits and PA at baseline and follow-up are summarised in Table 1.

The results showed no significant intervention effects for the following behaviours: daily breakfast consumption (p = 0.49), consumption of soft drinks (p = 0.22), fruit consumption (p = 0.52) and consumption of sweet and savoury snacks (p = 0.31). In addition, no significant time by condition interaction effect was found for active transport (p = 0.70) or for sports participation (p = 0.25). A trend towards significance (F = 2.95, p = 0.07) was found for the time effect of sports participation; the mean minutes of sports participation increased in both the intervention (from 158.02 ± 77.86 to 168.87 ± 69.73) and control (from 167.82 ± 67.45 to 170.99 ± 67.90) groups. Furthermore, the data revealed a trend towards significance for the time by condition interaction effect of water (p = 0.09) and vegetable consumption (p = 0.07). Reported water consumption increased from baseline to follow-up in the intervention group, whilst it remained the similar in the control group. An increase in reported vegetable consumption was noted in both groups, but there was a greater increase in the intervention group.

Regarding the psychosocial correlates of a healthy diet and PA, no significant time by condition interaction effects were observed for attitude towards having a daily breakfast (p = 0.82), attitude towards fruit consumption (p = 0.32), attitude towards water consumption (p = 0.62), attitude towards less sweet and savoury snack consumption (p = 0.86) and attitude towards PA (p = 0.36). Significant intervention effects were found for a positive attitude towards less soft drink consumption (p < 0.001), self-efficacy for having a daily breakfast (p = 0.003) and attitude towards vegetable consumption (p = 0.004). For pupils in the intervention group, attitude towards less soft drink consumption increased from baseline to follow-up, whilst it remained the similar for pupils in the control group. Self-efficacy towards daily breakfast increased in both groups, although the increase in the intervention group was significantly larger. A positive attitude towards vegetable consumption increased from baseline to follow-up in the intervention group and decreased in the control group. A trend towards a significant time by condition interaction effect was found for self-efficacy for meeting the PA guideline (p = 0.09). In the control group, self-efficacy for meeting the PA guideline was similar at baseline and follow-up, while it increased between baseline and follow-up in the intervention group.

### Process evaluation

The start and end clinics received a largely positive assessment from the 92 pupils who completed the process evaluation. The themes that were most commonly discussed in class were breakfast and vegetable consumption. More than half of the pupils said they saw the video messages and letters from the professional football players and the majority of the pupils assessed this contact as positive. With a mean score of 7.82 out of 10 the ‘Health scores!’ program was viewed positively overall (see Table 2).

**Table 2:** Process evaluation responses from pupils in the intervention group

**Questions and responses from intervention group pupils (n = 92)**	
*What did you think of the activities during the start clinic?*	
Very nice	49 (62%)
Nice	18 (23%)
Neutral	9 (11%)
Not very nice	1 (1%)
Not nice at all	2 (3%)
*Give an overall evaluation of the start clinic. (scale 1-10)*	
Mean score (SD)	
7.81 (2.34)	
*Which themes were discussed in class?*	
	(times reported)
Breakfast	64
Vegetables	52
Fruit	12
Healthy snacks	8
Physical activity	8
Other (open ended)	2 (food in general, water)
*Did you see the video messages and newsletters from the professional football players?*	
Yes	54 (59%)
No	37 (41%)
*What did you think of the video messages and newsletters from the professional football players?*	
Very nice	26 (39%)
Nice	26 (39%)
Neutral	11 (17%)
Not very nice	2 (3%)
Not nice at all	1 (1%)
*Would you have preferred more contact with the professional football players?*	
Yes	37 (54%)
No	19 (27%)
No opinion	13 (19%)
*What did you think of the activities during the end clinic?*	
Very nice	45 (56%)
Nice	22 (28%)
Neutral	8 (10%)
Not very nice	1 (1%)
Not nice at all	4 (5%)
*Give an overall evaluation of the end clinic. (scale 1-10)*	
Mean score (SD)	
7.32 (2.50)	
*Give an overall evaluation of the project ‘Health scores!’ (scale 1-10)*	
Mean score (SD)	
7.82 (2.38)	

## Discussion

The present study examined the effectiveness of the ‘Health scores!’ program, an intervention which uses professional football players to promote a healthy lifestyle in socially vulnerable children and adolescents. In line with the ELM [20], peripheral information processing was addressed combined with central information processing to communicate with this hard to motivate target population. The findings indicate that the intervention was mainly successful in increasing psychosocial correlates of a healthy diet and PA. Increases were found in self-efficacy for having a daily breakfast and towards reaching the PA guideline, the program also improved pupils attitudes towards less soft drinks and eating more vegetables. Furthermore, marginal evidence (p < 0.10) was found for an increase in vegetable consumption and water consumption. Similar to the Dutch study [25], which used a non-controlled design, no intervention effect was found for breakfast consumption or sweet and savoury snack consumption. In contrast to the Dutch study, no significant effect for soft drink consumption was observed. For sports participation only a significant time effect was found, with an increase in both groups.

One possible explanation for the significant time effect in sports participation could be that during leisure time children and adolescents in the intervention group discussed the ‘Health Scores!’ program with friends from the control group, resulting in contamination bias [32]. The lack of intervention effect for PA may be partly explained by the results from the process evaluation. These results indicated a problem with intervention delivery as it was noted that ‘PA’ as a topic was not often discussed during class and about 40% of the pupils reported not seeing the video messages and letters from the professional football players. This might indicate that the activities during the start and end clinic alone are not sufficient enough to change PA behaviour and must be complemented by a more structured school program. To minimize the barriers to participation, schools had a high degree of freedom in the implementation of activities and as a result some topics were not or scarcely discussed. The intervention did not affect active transport, this may be because factors associated with active commuting, such as environmental correlates and parental characteristics were not addressed [33].

For a healthy diet, positive intervention effects on behavioural correlates did not result in behaviour change, this may be because the intervention did not continue for long enough to influence behaviour. The lack of behavioural change could also be because of the lack of parental involvement in the intervention. It was previously found that through mechanisms of role modelling, development of attitudes and food availability at home, parents play an important role in the development of healthy eating habits of their children [34,35]. However, partly because of the language barrier, engaging parents of socially vulnerable children in interventions remains challenging.

Some psychosocial correlates did not change during the intervention, this might be due to ceiling effects. The mean attitude towards eating a daily breakfast, fruit consumption and PA was already very high at baseline, so the scope for achieving intervention effects was limited.

Analysis of the process data revealed that the activities during both the start and end clinics were well received by the pupils. The majority (78%) of the pupils assessed the contact with the professional football players as very positive. More than half of the pupils indicated they would have liked to have more contact with the footballers. However, it was not possible to determine whether pupils want more contact because they liked the educational messages or whether they just want to see their sports heroes more often. These findings, alongside the overall positive evaluation of the intervention suggest that using professional football players to promote health behaviours appeals to socially vulnerable children and adolescents. In the Dutch evaluation study [25] the focus was only on outcome evaluation. As such, it was impossible to make any comparison.

Most studies on information processing and source characteristics are embedded within advertising and consumer behaviour [27,28]. To our knowledge, only one study has reported the effects of using a credible source and positive message framing on exercise intentions and behaviours in college students [21]. The present study adds to this research by indicating that professional football players may be a credible source to promote a healthy diet and PA. Moving away from a purely cognitive approach and providing information through peripheral cues such as role models seems to be a good way to address the hard-to-reach populations of socially disadvantaged children and adolescents.

The intervention was implemented by local practitioners and not by researchers resulting in some methodological weaknesses such as the non-random assignment of pupils to the intervention and control group. Although control schools were matched to intervention schools, a number of baseline differences were observed between both groups. However, these baseline differences were taken into account by controlling for these variables in all analyses. Furthermore, because of the limited number of pupils from the intervention group that filled out a questionnaire the full impact of the intervention cannot be estimated. Intervention effectiveness could not be examined in girls due to the limited number of girls in the intervention group. The participants’ perceptions, involvement and knowledge of football was not assessed prior to the study. This is a further weakness because children from certain ethnic groups may not be familiar with football and may not consider football players heroes or role models. SES of the participants could not be calculated as more than half of the participants did not know the highest educational qualification achieved by their father. The current study did not incorporate a process evaluation among the teachers. This would have resulted in a more complete insight into the implementation of the intervention. Researchers and teachers were not trained to assist with the self-completion of the participants’ questionnaire, which could have introduced some bias. Although valid and reliable measures were used in this study, using self-report increased the likelihood that socially desirable responses were given. Despite the limitations, this study adds to the evidence on the effectiveness of health promotion programs delivered through football clubs in socially vulnerable children and adolescents.

## Conclusions

The study suggests that the combination of a school program with the use of professional football players to promote a healthy lifestyle is a promising strategy that appeals to socially vulnerable children and adolescents. However, the results are based only on boys and as boys have greater predilection for football than girls, further research on the use of football players to promote a healthy diet and PA in girls is needed. Moreover, future research could focus on gaining insights into whether professional football players are perceived as heroes by children from different ethnic backgrounds and whether a coaching approach for a longer period of time can achieve larger intervention effects.

## Abbreviations

PA: Physical activity; SES: Socio-economic status; ELM: Elaboration Likelihood Model; PE: Physical Education; FFQ: Food Frequency Questionnaire; FPAQ: Flemish Physical Activity Questionnaire.

## Competing interests

The authors declare they have no competing interests.

## Authors’ contributions

VD contributed to the design of the study (in consultation with the Football^+^ Foundation), analysed the data, led the writing of the paper and wrote the manuscript. IDB, LM, GC and KDC participated in the design of the study, helped to interpret the data and to draft the manuscript. KDM, JS, JL, and HB provided feedback on the manuscript. All authors read and approved the final manuscript.

## Pre-publication history

The pre-publication history for this paper can be accessed here:

http://www.biomedcentral.com/1471-2458/14/457/prepub
